# Non-local Schrödinger diffusion model reveals mechanisms of critical brain dynamics

**DOI:** 10.1016/j.xcrp.2025.102936

**Published:** 2025-11-19

**Authors:** Gustavo Deco, Yonatan Sanz Perl, Morten L. Kringelbach

## Main text

(Cell Reports Physical Science *6*, 102663; July 16, 2025)

In the originally published version of this article, Yonatan Sanz Perl was the corresponding author. This has been changed to Morten L. Kringelbach at the request of the authors, to reflect the role of Dr. Kringelbach as senior author on the article. All authors consent to this change.

